# Influence of surface pretreatments on the repair bond strength of 3D-printed resin used for permanent restorations: an *in vitro* study

**DOI:** 10.3389/fdmed.2026.1821223

**Published:** 2026-07-24

**Authors:** Ali Robaian

**Affiliations:** Conservative Dental Sciences Department, Prince Sattam Bin Abdulaziz University, Al-Kharj, Saudi Arabia

**Keywords:** adhesion, surface treatments, dental restoration repair, shear strength, three-dimensional printing

## Abstract

**Introduction:**

3D-printed permanent resin-based materials have recognized as practical options for permanent restorations because of their mechanical properties, aesthetic performance, and ability to work with digital workflows. There is a lack of studies on identifying the appropriate surface pretreatments to improve repairability of 3D-printed permanent resin. Accordingly, this study assessed how different surface pretreatment conditions impact the repairability of 3D printed permanent resin utilized for definitive restorations.

**Methods:**

Sixty cylindrical specimens from 3D-printed permanent resin (10 mm × 3 mm) were categorized according to their surface pretreatments into five groups after being subjected to 5000 thermocycles. The groups were as follows: Control (C), with no surface pretreatment; Diamond Bur (DB), in which the surfaces were roughened using a diamond bur; Sandblasting (SB), in which the surfaces were treated with airborne particle abrasion; Diamond Bur + Scotchbond Universal Adhesive (DB + SBU), in which diamond bur surface roughening was followed by the application of adhesive; and Sandblasting + Scotchbond Universal Adhesive (SB + SBU), in which airborne particle abrasion was followed by the application of adhesive. Onto the prepared CAD/CAM surfaces, a nanohybrid repair resin composite was packed. Subsequently, SBS and the mode of failure were conducted. Data were statistically analyzed using one-way analysis of variance, followed by Tukey's *post hoc* multiple comparison test at a *p* < 0.05.

**Results:**

The SB + SBU group had the highest mean repair SBS value (17.45 ± 2.78), whereas the C group demonstrated the lowest SBS value (10.32 ± 2.56). The DB + SBU group exhibited a significantly higher SBS (*p* < 0.001) in comparison to the control group. No statistically significant difference was observed between the DB and SB groups. Adhesive failure were predominantly observed in the control group. Cohesive failures were observed in the SB + SBU and DB + SBU groups, while mixed failures were observed in all groups except control groups.

**Conclusions:**

Within limitations of this study, roughening with diamond bur can effectively improve repair bond strength of 3D-printed permanent resin without a determinable effect of air particle abrasion. Additionally, applying a bonding agent after mechanical surface roughening improve the repairability of the 3D printed resin utilized for permanent restoration

## 1 Introduction

The advancement of CAD/CAM technology has enabled the production of various dental prostheses through three-dimensional printing methods. The incorporation of 3D-printing in dental treatment has considerably broadened the range of indirect restoration fabrications. This is because it has enabled faster production and improved economic efficiency in comparison to the conventional subtractive manufacturing methods (1, 2). 3D-printed permanent resin-based materials have recognized as practical options for permanent restorations because of their mechanical properties, aesthetic performance, and ability to work with digital workflows (3).These materials are primarily composed of cross-linked methacrylate-based polymer network reinforced with inorganic fillers and activated by photo initiators, making them designed for long-term intraoral use.

Despite the advantages of permanent 3D-printed resins, its ability to be repaired intraorally following chipping, cracking, or edge failure, is a major concern that could impact their durability (4, 5). In such clinical scenarios, intraoral repair procedures are typically selected as a more economical and conservative option than replacing the restoration entirely. The efficacy of composite resin repair on aged 3D-printed resin substrates is affected by various interrelated parameters, notably surface conditioning methods and the choice of adhesive systems (5–7). The interfacial bond between the newly applied resin layers and the existing restoration tends to deteriorate over time, necessitating special surface treatment for the repair (8, 9). Appropriate surface treatments are essential for establishing a durable bond and preventing repeated fracture and repair cycles (10, 11).

The interfacial bond between newly applied resin layers and the existing restoration tends to degrade over time, making specific surface treatment essential during repair procedures (8, 9). Hence, effective surface treatment is essential for establishing a strong bond and reducing the likelihood of recurring fractures and repairs (10, 11).

Airborne particle abrasion is a commonly employed mechanical surface treatment method that improves the adhesive strength of polymer materials. This procedure involves the application of alumina particles to the surface, which disrupts the polymer matrix and filler, resulting in a textured surface that boosts both the surface area and mechanical interlocking (12, 13). In addition, universal adhesives may enhance repair bonding by promoting chemical interaction between the aged restoration and the repair composite. These adhesives contain functional monomers, such as 10-methacryloyloxydecyl dihydrogen phosphate (10-MDP), and silane coupling agents, which improve substrate wettability and chemical bonding to exposed filler particles after surface roughening (11). When it comes to intraoral repair procedure, airborne particle abrasion isn't always a practical or viable option. Accordingly, there is a need to explore alternative surface treatment approaches capable of achieving repair bond strength comparable to the airborne particle abrasion. Methods include adhesive application, abrasion using silicon carbide paper, or abrasion with diamond burs (14–17).

There is a lack of studies on identifying the appropriate surface pretreatments to improve the repairability of 3D printing permanent resins. As a result, this study investigated the impact of various surface pretreatments on the reparability of 3D printed permanent resins utilized for permanent restoration. The null hypothesis examined was that various surface pretreatments would not influence the repair bond strength of the 3D printed permanent resin.

## 2 Methodology

### 2.1 Materials

A single commercially available 3D-printed permanent resin and one universal adhesive system were selected for evaluation. Detailed information regarding their composition and manufacturers is provided in Table 1.

**Table 1:** Materials used in the study.

Product (Composition)	Manufacturer	Lot. No.
3D printed Resin Post Saremco print CROWNTEC Shade A3	Bisphenol A polyethylene glycol diether dimethacrylate (BisEMA, 50-<75%), methyl benzoylformate (1-<5%), and diphenyl(2,4,6 trimethylbenzoyl)phosphine oxide (1-<5%) -Saremco Dental AG, Rebstein, Switzerland	E394
Single Bond Universal	-MDP, dimethacrylate resins, HEMA, Vitrebond copolymer, filler, thanol, water, initiators, silane -3M ESPE, St. Paul, MN, USA	Z01 × 78
Filtek Supreme XTE	-Matrix: Bis-GMA, UDMA, TEGDMA, PEGDMA, Bis-EMA. -Filler load: 63.3 vol%, 78.5 wt% -3M ESPE, St. Paul, MN, USA	N470318

The sample size was estimated using G*Power software (version 3.1.9.7). Using a previously reported effect size of 0.365 (12), a significance threshold of *α* = 0.05, and a statistical power of 80%, a total of sixty specimens were prepared and randomly allocated into five groups, with twelve specimens assigned to each group (*n* = 12).

### 2.2 Specimens’ preparation

Sixty disc-shaped specimens, each measuring 10 mm in diameter and 3 mm in thickness according to ISO 4049 for polymer-based restorative materials, were produced using a digital light processing (DLP) resin-based 3D printer (ASIGA Composer UV, Sydney, NSW, Australia). The specimens were digitally designed using CAD software (Meshmixer 3.5.0; Autodesk, San Francisco, CA, USA) and exported as standard tessellation language (STL) files and then transferred to desktop 3D printer (Asiga Max UV printer, Sydney, NSW, Australia) to produce 3D printed specimens. The printing procedures were carried out according to manufacturer's instructions, as previously outlined (18). Before specimen fabrication, the printer underwent calibration and verification to ensure dimensional accuracy and consistent processing conditions. According to the manufacturer's guidelines, the printing parameters were set as follows: the printing layer thickness was selected as 50 μm with an exposure time of 5 s per layer. A layer thickness of 50 μm was selected according to the manufacturer's recommendations for the resin–printer system used, ensuring adequate dimensional accuracy and surface quality. The material was printed with layer orientation parallel to longitudinal axis of the specimens. A digital caliper was utilized to ensure the accuracy of the specimen dimensions. Following fabrication, specimens underwent thorough visual and dimensional inspection to ensure compliance with the established design specifications. Surface integrity was assessed using a stereomicroscope at 10× magnification, with specimens showing surface cracks or structural defects being excluded from the study (12, 18).

After fabrication, the specimens were removed and ultrasonically cleaned in 90% isopropyl alcohol for 5 min to remove residual uncured resin. Post-polymerization was conducted for 7 min using a dedicated light-curing unit (Otoflash G171-N2, NK Optik GmbH, Baierbrunn, Germany) following air-drying. The unit operates within a wavelength range of 300–700 nm, delivering high-intensity xenon light flashes, with two flashes of 1500 light pulses each, under nitrogen atmosphere, to ensure complete polymerization. Specimens were positioned centrally within the curing chamber at a standardized distance to ensure uniform light exposure. All specimens were post-cured under identical conditions to ensure consistency. All specimens underwent standardized surface finishing through sequential silicon carbide abrasive papers, utilizing grits from 600 to 2,000, while maintaining continuous water irrigation. They were subsequently rinsed with deionized water for three minutes to achieve a uniform standard. All specimens underwent 5,000 thermocycles (Thermocycler, SD Mechatronik, GmbH, Feldkirchen-Westerham, Germany) at temperatures between 5 and 55 °C, featuring a dwell duration of 30 s and a transfer time of 10 s. Following aging, specimens were categorized into five groups (*n* = 12/group) based on the conditions of the surface pretreatments.

### 2.3 Surface pretreatments and grouping

Specimen were mounted in an acrylic resin, ensuring that one surface remained accessible for further treatment procedures. The bonding interface was precisely delineated by adapting a polyethylene adhesive sheet with a circular window (5-mm in diameter) and positioned in the center to the exposed surface.

Specimens were categorized into five groups based on their surface pretreatments: Groups C, DB, DB + SBU, SB, and SB + SBU. Group C had no surface pretreatments. Group DB; Surfaces were subjected to grinding using a medium grit tapered round-end diamond bur (856, Komet Dental GmbH, Lemgo, Germany; grit size: 107–126 μm) operated at 45,000 rpm under water cooling for 8 s (15, 19). Group SB: Surfaces were abraded with 50 μm aluminum oxide particles (LEMAT NT4, Wassermann, Dental-Maschinen GmbH, Hamburg, Germany) for 10 s at 10 mm distance and 0.55 MPa pressure, followed by 20 s of air drying (15, 18). Regarding the DB + SBU and SB + SBU groups, surfaces of the specimens were subjected to the same procedures as in groups DB and SB, respectively with addition of Scotchbond Universal Adhesive (3M ESPE, St. Paul, MN, USA). All surface pretreatment procedures were performed by a single calibrated operator to minimize operator-related variability. To control for bur wear, diamond burs were replaced at standardized intervals (5 times usages), ensuring consistent cutting efficiency and uniform surface treatment across all specimens.

### 2.4 Repair procedures

A 6 mm circular split Teflon mold with a 2 mm thickness (7) was used to apply nanohybrid repair resin composite Filtek Supreme XTE (3M ESPE, St. Paul, MN, USA) to the 3D printed resin's treated surfaces in all groups. The mold was filled incrementally using 2-mm composite layers, with each layer light-cured separately. The bonding area was standardized to 5 mm in diameter using a polyethylene adhesive window. The 6 mm split Teflon mold was used only to facilitate composite placement and to ensure complete coverage of the bonding area. For DB + SBU and SB + SBU groups, a thin layer of Single Bond Universal adhesive was uniformly applied to the treated surfaces according to the manufacturer's instructions. The adhesive layer was air-thinned for 5 s, after which it was polymerized using light activation for 20 s.

### 2.5 SBS testing

The shear bond strength test was conducted using a universal testing machine (Model TT-B, Instron Corp., Canton, MA, USA) in accordance with DIN EN ISO 10477 (20). The bonded CAD/CAM polymer-resin composite assembly was accurately positioned in the machine's lower jaw to align with the direction of shear force, ensuring parallelism. The upper movable compartment of the testing apparatus firmly attached to a mono-beveled chisel stainless-steel rod and was precisely aligned at the interface (15). A compressive force was applied at a crosshead speed of 0.5 mm/min. The shear bond strength (SBS) in megapascals (MPa) is calculated by dividing the shear force at fracture, measured in Newtons (N), by the bonded surface area (5 mm in diameter), expressed in square millimeters (mm^2^). The digital caliber was utilized to determine the bonded surface area.

### 2.6 Scanning electron microscopy surface topography

The surface topography of 3D-printed material was evaluated before and after surface treatments. For each group, three additional specimens (10 mm × 10 mm × 1 mm) were developed as previously described. The specimens were then cleaned with 96% ethanol in an ultrasonic bath for two minutes prior to air-drying. For the qualitative analysis, specimens were attached to metallic stubs, subjected to gold sputter-coating, and examined using a 2000×SEM (Jeol-JSM-6510, Tokyo, Japan) (12, 14).

### 2.7 Mode of failure

The fractured specimens’ were examined using a 40-x optical stereomicroscope (Olympus SZ61, Tokyo, Japan) to analyze the mode of failure subsequent to debonding. The failure was classified into three distinct categories: cohesive failure occurring within the resin composite, adhesive failure at the interface, and a mixed type of failure (18).

### 2.8 Statistical analysis

The homogeneity of variance was examined using Levene's test (*α* = 0.05), and intergroup normality was determined using a Shapiro–Wilk test. A one-way analysis of variance (ANOVA) with Tukey's test for multiple comparisons (*α* = 0.05) was used to examine the variations among the groups.

## 3 Results

**Figure 1:**
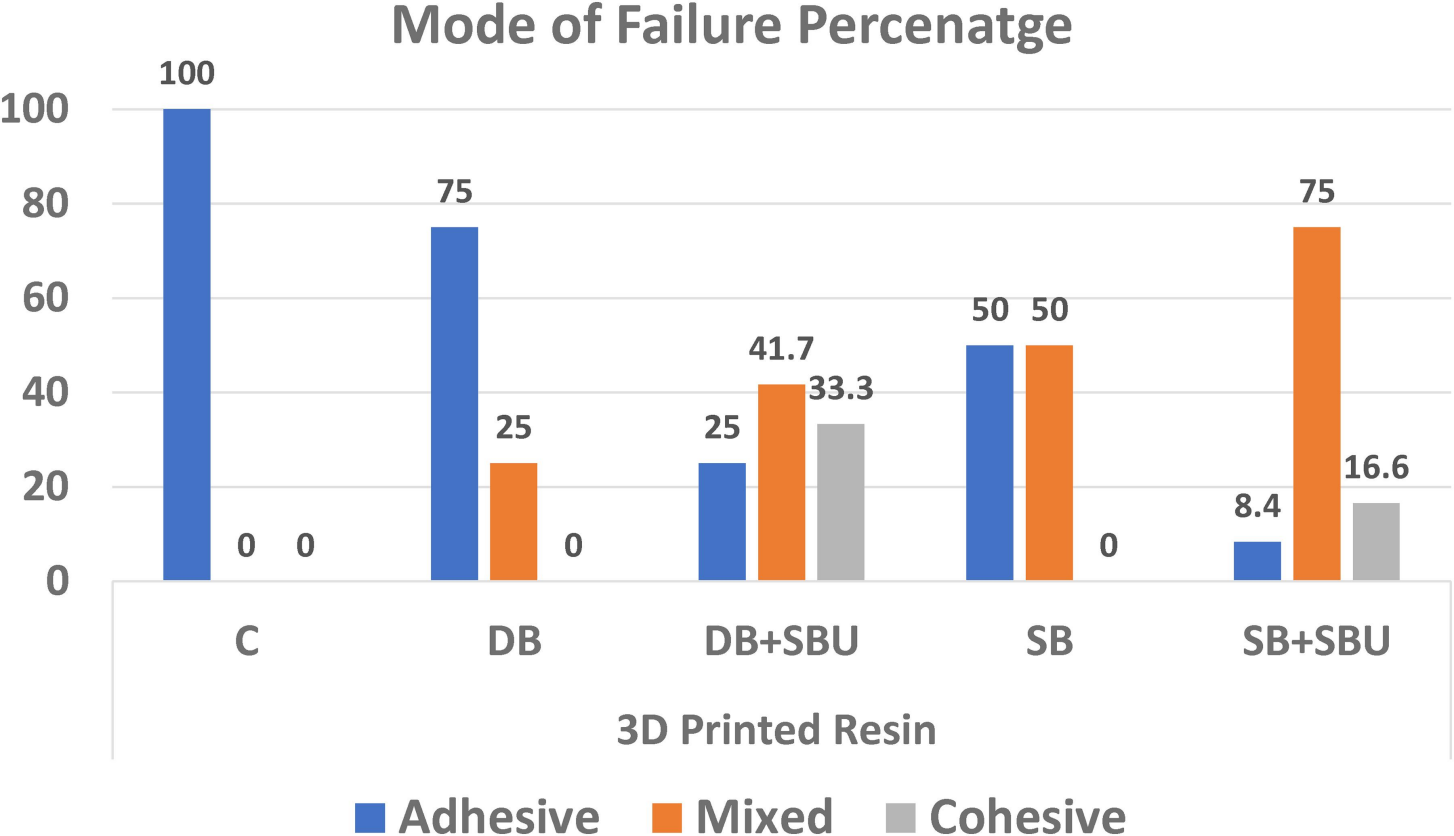
Mode of failures for the different groups.

The mean repair shear bond strength (SBS), together with the distribution of failure modes across the experimental groups, are illustrated in Table 2 and Figure 1. Following confirmation of normal data distribution, comparative analysis was undertaken using one-way ANOVA to detect overall group differences. *post hoc* multiple comparisons were subsequently performed using Tukey's test, which identified statistically significant differences between the surface pretreatment methods (*p* < 0.05).

**Table 2:** Shear bond strength and modes of failure for the different groups.

Groups	Repair Bond Strength	Mode of Failure (Percentage)		
		Adhesive	Mixed	Cohesive
C	10.32 ± 2.56 c	100	0	0
DB	13.45 ± 2.78 b	75	25	0
DB + SBU	15.88 ± 2.78 a	25	41.7	33.3
SB	11.85 ± 2.78 b	50	50	0
SB + SBU	17.45 ± 2.78 a	8.4	75	16.6

Mean values represented with same lowercase letters (row) are not significantly different according to Tukey's test (*P* > 0.05).

### 3.1 Repair shear bond strength

The highest mean repair SBS value (17.45 ± 2.78) was recorded for SB + SBU group, whereas the lowest SBS value (10.32 ± 2.56) was for C group. DB + SBU group recorded significantly higher SBS (*p* < 0.001) compared to control group. No significant difference (*p* = 0.68)where detected between DB and SB groups**.**

### 3.2 Scanning electron microscopy evaluation (SEM)

**Figure 2:**
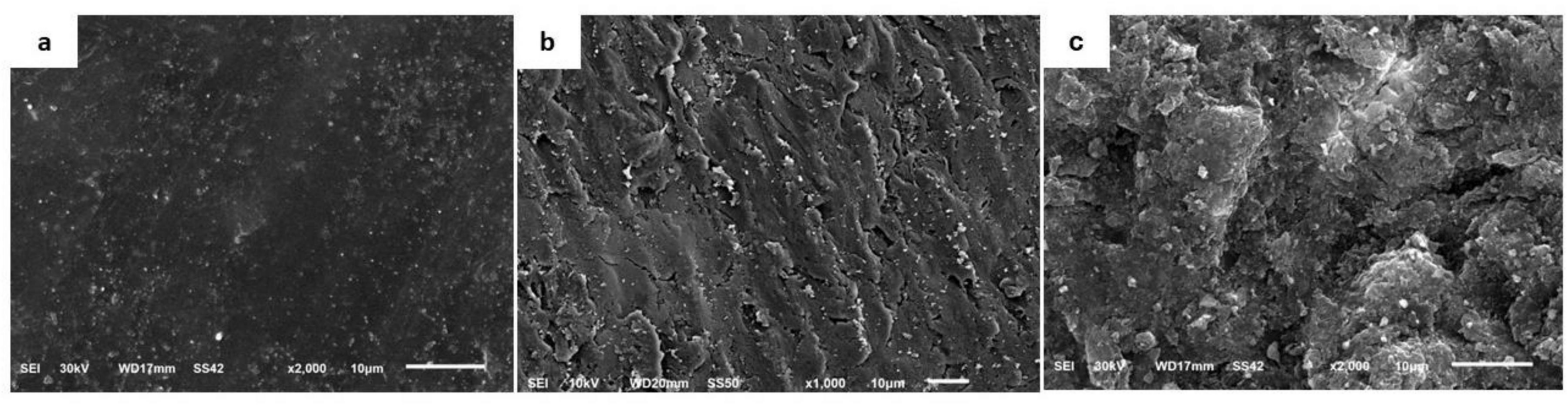
SEM micrographs of 3D printed permanent resin after different surface treatments (2000 x). **a**: control group (no surface treatment), **b**: diamond bur grinding group, and **c**: air particle abrasion group.

SEM micrographs of the treated 3D printed resin material are presented in Figure 2. SEM analysis revealed distinct surface morphologies depending on the applied surface treatment. The control group showed a smoother surface pattern than did the diamond bur and air particle abrasion groups. The diamond bur showed an irregular topography characterized by pronounced grooves. However, specimens subjected to air particle abrasion demonstrated a markedly roughened and more uniformly micro-textured surface, featuring micro-irregularities and sharp-edged depressions.

### 3.3 Mode of failure

Adhesive failure were predominantly observed in the control group. Cohesive failures were observed in the SB + SBU and DB + SBU groups, while mixed failures were observed in all groups except control groups. Failure type frequencies are given by group in Table 2 and Figure 1.

## 4 Discussion

The study's findings demonstrated a statistically significant impact of surface pretreatment protocols on repair bond strength (*p* < 0.05) of 3D-printed permanent resin. Among the tested groups, the application of a bonding agent following mechanical surface treatment resulted in the highest bond strength values. In contrast, air particle abrasion alone did not significantly improve bond strength compared to the control. Therefore, the null hypothesis, which stated that surface pretreatments would not affect repair bond strength, was rejected.

Repairing rather than replacing a restoration is a key therapeutic method in minimally invasive dentistry. Repairing a restoration has a clinical advantage over replacement, which requires more cavity preparation and higher treatment costs (21, 22). The durability of a repaired restoration is largely influenced by the strength and stability of the interface between pre-existing restoration and the newly placed resin composite. The interfacial bonding depends significantly on the surface pretreatment protocol applied to the aged restoration before repair (23).

Surface pretreatments, including acid etching, laser irradiation, aluminum oxide or silica-coated particles, and roughening with burs, have been shown to substantially improve micromechanical interlocking in previous studies (14, 15). Dental burs, are frequently chosen for surface roughening in routine clinical practice because of their accessibility and simplicity. Additionally, air abrasion was selected as the pretreatment method in this study due to its ability to deliver a standardized and reproducible roughening effect, which minimizes operator variability (24, 25).

Chemical pretreatments, including adhesive systems, are essential for achieving effective chemical bonding, alongside mechanical roughening (26, 27). This study assessed the interfacial bonding performance of repaired 3D-printed permanent resin using the shear bond strength (SBS) test. This method was chosen for its widespread acceptance as a simple and reliable *in vitro* technique for assessing bond strength, especially in samples with relatively large bonding areas (approximately 3–6 mm in diameter) (12, 14). Also, in this study, the specimens were aged through 5,000 thermal cycles simulating 6 months of clinical usage to produce aged substrate surfaces that replicate clinical oral circumstances.

Beher et al. (28) reported that a shear bond strength of 10 MPa at the interface between resin-based materials and the underlying substrate is considered clinically acceptable. In the present study, all mechanically roughened groups demonstrated bond strength values above 10 MPa, indicating that this surface treatment approach may be considered clinically acceptable.

This study found that air particle abrasion did not significantly enhance bond strength in comparison to the control group. In agreement with Yoshihara et al. (29), this surface treatment may compromise the resin matrix, expose filler particles in 3D-printed resin polymers, and create sites of stress concentration. In line with our results, Lim et al. (30) and Lee et al. (12) also reported that 3D-printed resin's bond strength was not considerably enhanced by air particle abrasion. As a result, when utilizing air particle abrasion on 3D-printed resins, caution is recommended and alternative surface treatments should be considered to improve bond strength without sacrificing their properties. While previous studies have suggested that air abrasion may alter the resin surface, such effects were not directly evaluated in the current study. Therefore, these findings should be interpreted with caution, and further investigations are required to clarify the underlying mechanisms. Conversely, roughening with a diamond bur significantly enhanced the bond strength through creating macro- and micro-retentive areas, which in turn increased the surface area for enhanced adhesion (15, 26). Our study findings are in line with weignad et al. (15) and Soliman et al. (19) who reported improvement of the bond strength after roughening with diamond burs.

In this current study, application of bonding agent significantly improve the repair bond strength in DB + SBU (15.88 ± 2.78) and SB + SBU (17.45 ± 2.78) groups compared to DB (13.45 ± 2.78) and SB (11.85 ± 2.78) groups. The bonding agent used in this study comprises 10-MDPI. It improves chemical bonding at the adhesive interface and promote chemical interaction with the substrate through ionic bonding and improved wettability. Additionally, it increases the SBS by incorporating silane which form chemical bond with the exposed fillers particle and increase surface energy of substrate and hence improve adhesion (12). Our findings aligned with of Lee et al. study (12) who reported that applying bonding aging after surface treatment improve repair bond strength. In contrast, several studies have found that using a bonding agent had no significant effect on the bond strength of CAD/CAM resin materials. As a result, additional studies are needed to assess the impact of various bonding agents on repair bond strength.

The mode of failures are consistent with bond strength findings. The control group experienced more adhesive failures, indicating weaker interfacial adhesion. In contrast, DB + SBU and SB + SBU demonstrated a greater proportion of mixed and cohesive failures, indicating better interfacial bonding and enhanced micromechanical retention. These findings are consistent with earlier studies that reported improved interfacial performance of single bond universal when combined with mechanical pretreatments procedures (31, 32).

The results of this investigation revealed that diamond bur roughening significantly enhances the bond strength of 3D printed resin, without any determinable effect of air particle abrasion. The current study has limitations: Only one type of 3D-printed resin and one universal adhesive system were evaluated; therefore, the findings cannot be generalized to other materials or adhesive formulations. In addition, only one artificial aging protocol (5,000 thermocycles) was used and repair bond strength was assessed immediately after repair without evaluating long-term bond durability. Although SBS testing is widely used and practical, microtensile bond strength testing may provide more uniform stress distribution and might be considered in future studies. Surface characterization was limited to qualitative SEM analysis without quantitative roughness measurements. Furthermore, additional research is required on various chemical surface treatments and the application of different adhesive systems. Also, future studies incorporating quantitative surface profilometry are recommended to provide a more comprehensive evaluation. Furthermore, well-designed controlled clinical trials with standardized protocols are essential to fully assess long-term clinical performance.

## 5 Conclusion

Within the limitations of this study, diamond bur surface roughening appeared to be a more promising strategy for enhancing the repair bond strength of 3D-printed resin, since it improved adhesion without the potentially negative surface effects associated with air particle abrasion. Additionally, applying a bonding agent after mechanical surface roughening improve the repairability of the 3D printed resin utilized for permanent restoration.

## Data Availability

The original contributions presented in the study are included in the article/Supplementary Material, further inquiries can be directed to the corresponding author.
